# Dielectric Relaxation Behavior of BTO/LSMO Heterojunction

**DOI:** 10.3390/nano11051109

**Published:** 2021-04-25

**Authors:** Guoqiang Song, Yuanyuan Zhang, Sheng Li, Jing Yang, Wei Bai, Xiaodong Tang

**Affiliations:** 1 Key Laboratory of Polar Materials and Devices, Ministry of Education, Department of Electronic Science, East China Normal University, Shanghai 200241, China; 51181213012@stu.ecnu.edu.cn (G.S.); 51181213007@stu.ecnu.edu.cn (S.L.); jyang@ee.ecnu.edu.cn (J.Y.); wbai@ee.ecnu.edu.cn (W.B.); 2 Collaborative Innovation Center of Extreme Optics, Shanxi University, Taiyuan 030006, China

**Keywords:** BaTiO_3_, manganese, Maxwell-Wagner effect, magnetoelectric coupling

## Abstract

The BaTiO_3_ (BTO)/La_0.7_Sr_0.3_MnO_3_ (LSMO) magnetoelectric composite films were prepared by sol-gel method on STO (001) substrates. The heterojunction has highly preferred orientation and exhibits well ferroelectric properties with perfect hysteresis loops and microscopic polarization switch behaviors. The most interesting thing is the abnormal dielectric relaxation phenomenon in the dielectric spectra at high frequency range and around the phase transition temperature of LSMO. By analyzing the resistance properties of LSMO films, it is indicated that charge-based interfacial coupling, Maxwell-Wagner effect due to the JT polaron and fast resistivity rise in LSMO layer is the main reason. This work emphasizes the crucial role of resistivity exchanges and of carrier accumulation at interfaces for the application of magnetoelectric heterojunction.

## 1. Introduction

The multiferroic heterojunction composed of single-phase ferroelectric (FE) materials and ferromagnetic (FM) materials has great potential applications in next-generation information processing and storage devices, such as memory computer read heads, sensors, and multi-state memories [1,2,3,4]. Most recently, FE/FM heterostructures with ABO_3_ perovskite structure are the most attractive materials for practical implementation of multiferroic studies of magnetoelectric coupling. BaTiO_3_ (BTO)/manganite heterojunction attracts much scientific and technological attention due to the excellent magnetoelectric coupling effect. The BTO/manganite can constitute a multiferroic tunnel junction which exhibits four or even eight resistance states in a single memory unit cell which can open a new pathway toward high-density nonvolatile memory [1,2]. The tunnel electroresistance can be manipulated by a ferroelectrically induced phase transition in the inserted manganite layer at the interface. A number of theoretical and experimental studies attributed magnetoelectric coupling to the interplay between changes in magnetization and accumulation/depletion states at the ferroelectric-ferromagnetic interfaces. Usually, the dominating intrinsic magnetoelectric coupling mechanisms are strain elastic coupling in piezoelectric/magnetostrictive systems, hybridization between 3d orbitals of interfacial atoms in FM metal/FE superlattices, and coupling of magnetic spins and electric dipole in FM nanoparticle/FE nanocomposites [5,6,7]. On the other hand, an extrinsic mechanism driven by localization of free charges at the interfaces was yielded by the combination of magnetoresistance (MR) and Maxwell–Wagner (MW) interfacial relaxation without intrinsic ME coupling [4,8,9]. Actually, it is experimental proved that the magnetoelectric coupling in BTO/doped-manganite heterojunctions can be regulated by the external electric and magnetic field [10,11,12,13]. In particular, it is speculated that the physical properties especially the dielectric properties in BTO/doped-manganite heterojunction can also be tuned by the temperature dependent self-owned phase transition in manganite.

In this work, BTO served as a ferroelectric constituent of the bilayer not only due to the archetype of ferroelectrics but also the sensitive physical properties [14,15]. The manganite La_1−x_A_x_MnO_3_ (A = Ca, Sr, or Ba), which is strongly correlated system with perovskite structure, has a strong interplay between electron transport, magnetism, and crystal lattice distortions and a rich carrier-density-temperature phase diagram. Among them, La_0.7_Sr_0.3_MnO_3_ (LSMO) not only exhibits ferromagnetic properties with high Curie temperature but also a metallic conduction at room temperature which can be served as the bottom electrode for the ferroelectric and dielectric properties measurements [16,17]. The structural, ferroelectric, and dielectric properties are systematically studied in this study. The dielectric relaxation phenomenon was observed in the temperature and frequency dependent dielectric properties of BTO/LSMO thin films. By analyzing the activation energy and the electrical transport properties of LSMO, it is proved that the dielectric relaxation phenomenon is closely related to the MW effect of LSMO thin films.

## 2. Materials and Methods

The BTO/LSMO heterostructures were prepared on SrTiO_3_ (STO) (100) single crystal substrate by sol-gel method. Lanthanum acetate (La(CH_3_COOH)_3_), strontium acetate (Sr(CH_3_COO)_2_), and manganese acetate (C_6_H_9_MnO_6_·2H_2_O) were dissolved into a solvent containing acetic acid (CH_3_COOH), deionized water and acetylacetonate to prepare the precursor solution. Barium carbonate (BaCO_3_) and titanium isopropoxide (C_12_H_28_O_4_Ti) were used as the sources for the precursor solution. The rotation speed and the spin time were at 5000 rpm and 30 s, respectively. After coating each layer, the film was fired at 200 °C for 180 s, then pyrolyzed at 400 °C for 180 s, and finally annealed at 850 °C for 600 s. Repeat the sinter process several times, and we finally get the BTO/LSMO composite film. The thicknesses of both LSMO and BTO layers are about 100 nm. The phase purity and crystal structure of the films were measured *θ*-2*θ* by high-resolution X-ray diffraction (HRXRD, Bruker, D8 Discover, Karlsruhe, GER). Ferroelectric material test system (precision premier II, Radical, Albuquerque, NM, USA) was used to characterize the ferroelectric properties. Piezoelectric atomic force microscopy (PFM, cypher, Asylum Research, Santa Barbara, CA, USA) was used to characterize the microscopic ferroelectric domain properties. Dielectric properties were measured using a precision LCR meter (Agilent E4980A, Palo Alto, CA, USA) mounted on physical property measurement system (Quantum Design, PPMS-9T, San Diego, CA, USA) over the frequency range of 20 Hz–1 MHz within the temperature range of 10–400 K.

## 3. Results and Discussion

**Figure 1:**
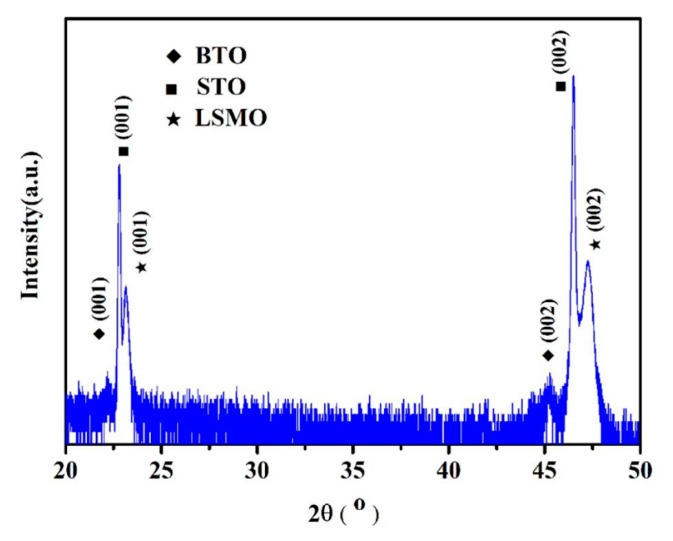
*θ*–2*θ* patterns of BTO/LSMO films grown on (001) SrTiO_3_ at room temperature.

Figure 1 shows the *θ*-2*θ* patterns of BTO/LSMO bilayer composite films grown on STO (001) substrate. It is observed only the (00*l*) diffraction peaks of films and the substrates, which shows that the films have strong crystallographic texture and highly preferred orientation. The corresponding angles of BTO and LSMO diffraction peaks are 47.22° and 45.41°. The lattice constants in the out-of-plane direction are 0.3846 nm and 0.3992 nm for LCMO and BTO, respectively. Compared with the bulk LSMO (a = 0.3864 nm) and BTO (a = 0.3993nm), it is found that the out of plane strain of LSMO thin films is 0.4%, while the lattice constant of BTO film is almost constant, indicating that it is almost relaxation.

**Figure 2:**
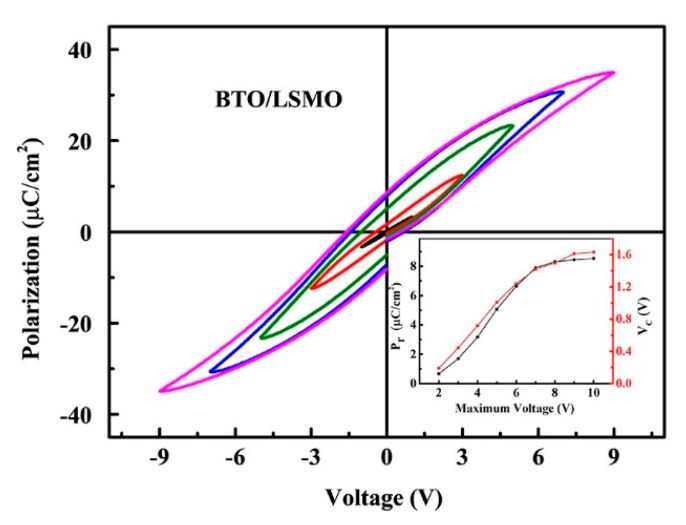
Hysteresis loops of BTO/LSMO films. The inset shows the relationship between coercive field and remanent polarization with the maximum electric field at room temperature.

Figure 2 shows the polarization hysteresis loops of Pt/BTO/LSMO at 1000 Hz with different maximum electric fields. The shapes of hysteresis loops are clear though they are not very slim at different voltages. The inset plots the applied voltage dependence of the remanent polarization (P_r_) and coercive voltage (V_c_) of the thin film. When the external electric field is below the coercive field, the coercive field and remanent polarization increase significantly with the increase of the electric field. After that, the change becomes smaller with the applied voltage, which indicates the saturation of the polarization hysteresis loops. The maximum remanent polarization and coercive field were 8.5 μC/cm^2^ and 1.6 V, respectively.

**Figure 3:**
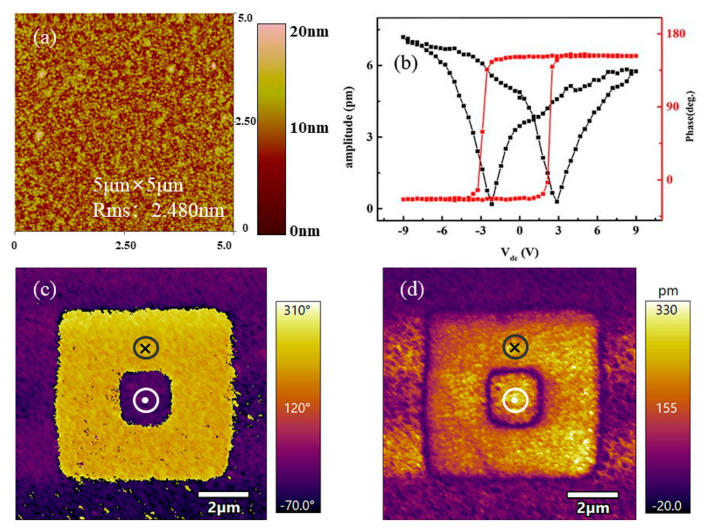
(**a**) Topography. (**b**) Off-field hysteresis loops of amplitude and phase. (**c**) PFM phase. (**d**) PFM amplitude for BTO/LSMO at room temperature.

The microscopic piezoelectric and domain properties were analyzed by PFM. Figure 3a shows the topography of film surface which is smooth, compact, and without obvious cracks. The butterfly amplitude loops and 180° phase flips when the amplitude is at a minimum were obtained in BTO/LSMO film, as shown in Figure 3b. The PFM hysteresis loop can be analyzed similarly with the macroscopic P-E loop in Figure 2. The shape of the 180° phase flips is similar to ferroelectric loop, while the voltage at a minimum amplitude (1.9 V) in the butterfly amplitude loops is almost the same with the coercive voltage (1.6 V) in Figure 2. Figure 3c,d show out-of-plane (OP) phase and amplitude of the corresponding area obtained after the central area poled at 10 V bias. Applying different voltages above the coercive voltage will lead to the polarization reversal to the direction of external electric field. It indicates that the samples have better remanent polarization ferroelectric can be reversed by applying different voltages and the as-grown BTO thin film has the upward self-polarization due to the same color with that of the +10 V poled area. It is mainly caused by the compressive epitaxial strain from the electrode and substrate, which can induce the upward self-poling [18,19,20].

**Figure 4:**
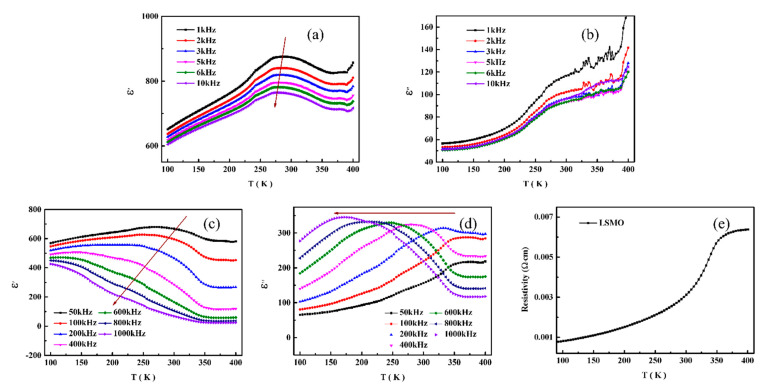
Temperature dependence of permiticity: (**a**) real and (**b**) imaginary parts at low frequencies; (**c**) real and (**d**) imaginary parts at high frequencies. (**e**) Temperature dependence of resistance of LSMO thin films.

The temperature dependences of dielectric properties of BTO/LSMO thin film at various frequency are shown in Figure 4a–d. It can clearly observe the abnormal phenomenon that the dependence of the real and imaginary parts of dielectric properties on temperature have a typical platform (270~300 K) which moves toward low temperature especially at the high frequency (>50 kHz). When the frequency is above 400 kHz, the platform disappears and e′ decreases gradually and tends to be stable at high temperature, accompanied by a relaxation peak of the imaginary part moves to low temperature with increasing frequency. The maximum magneto-induced effects on the polarization and dielectric properties occur within that temperature range which is related to the phase transition of LSMO, as shown in Figure 4e [10]. The resistivity of LSMO film increases fast in temperature range 270~300 K, and metal-insulator transition is around 350 K. It is proved that the dielectric relaxation behavior of BTO/LSMO observed at high frequency is closely related to the resistance of LSMO and Maxwell-Wangner (MW) effect [9,10]. Based on this phenomenon, intrinsic magnetoelectric coupling mechanisms, such as the strain elastic coupling, hybridization of interfacial atoms, and coupling of magnetic spins and electric dipole, can be excluded, and the charge-related interfacial effect may be the dominated factor for the temperature and frequency dependent magnetoelectric coupling effect [9]. The dielectric relaxation behavior of the heterogeneous interface of BTO/LSMO thin films mainly comes from the extrinsic heterogeneous inhomogeneity with grain boundary effect, the vibration of ferroelectric domain wall, and MW type dielectric relaxation [21,22]. As mentioned before, our BTO/LSMO films prepared by sol-gel method have highly preferred oriented, so the effect of grain boundary interface can be ignored. In addition, the phenomenon of dielectric relaxation caused by ferroelectric domain walls can also be ignored because it is normally observed occurring at much higher frequencies (>10^6^ Hz), and our films have upward self-poling and less domain wall, demonstrated by the PFM results. The LSMO have increasing resistivity with the increasing temperature, thermal carrier localized, and accumulated at the BTO/LSMO interface. This is consistent with the MW-type relaxation phenomenon. Therefore, we believe that the relaxation phenomenon observed in BTO/LSMO thin films may be due to MW effect with the carrier accumulation at the junction surface.

As the thicknesses of both LSMO and BTO layers are 100 nm, the local nanoscopic regions are formed with sufficient volume and thickness of LSMO, and the BTO/LSMO heterojunction consist of three layers: the BTO layer, the BTO/LSMO boundary layer, and the LSMO layer [23,24]. It means that the ferromagnetic LSMO layer containing pairs of Mn^3+^-Mn^4+^ ions in octahedra and electrons recharging due to the balance of the strong interactions of double exchange, Jahn-Teller effect and Coulomb repulsion. The temperature dependence of the resistance of LSMO layer is shown in Figure 4e. The hopping process of JT polaron may be responsible for the relaxation phenomenon. The relaxation dielectric spectra for frequencies above 50 kHz containing layers with different conductivities and permittivities can be influenced by not only by Maxwell-Wagner but also Debye relaxation relaxations at the BTO/LSMO interface. Further research should be done to clarify the physical mechanism.

**Figure 5:**
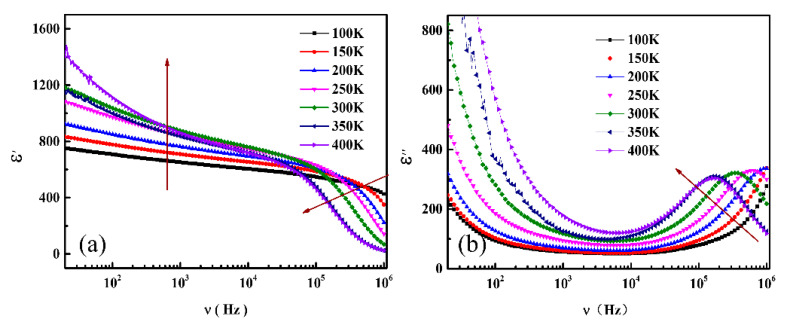
Frequency dependence of (**a**) real and (**b**) imaginary parts of dielectric constant at different temperatures.

Frequency dependent dielectric spectra of BTO/LSMO films at several temperatures is shown in Figure 5. It can be seen that the dielectric properties of the samples are obviously dispersed and very sensitive to temperature, especially in high frequency range. We need to analysis the system by the MW-type relaxation model. First, the inductance arises from the LSMO thin film and contacts loss can be neglected at the low frequency <1 MHz measurement. In a two-phase system, the relaxation time of heterojunction can be expressed as: t = *C*_1_*R*_2_, with *C*_1_ and *R*_2_ corresponding to the larger capacitance and the smaller impedance of the two substances, respectively [25]. In BTO/LSMO system, *C*_1_ usually does not change with temperature, but *R*_2_ is closely related to temperature which is the main factor in the study of dielectric relaxation. Compared to the resistivity of BTO ~10^12^ W cm, the resistivity of LSMO is much smaller ~10^−1^ W cm, so the relaxation behavior of this heterojunction is closely related to the resistivity of LSMO layer. When the temperature rises, quick increasing of resistivity of LSMO is observed in the temperature and then causes the dielectric relaxation.

## 4. Conclusions

In conclusion, BaTiO_3_/La_0.70_Sr_0.30_MnO_3_ (BTO/LSMO) bilayer magnetoelectric composite thin films were prepared by sol-gel method on STO substrates. XRD patterns show that the BTO/LSMO films have highly preferred oriented growth. The film shows good ferroelectric hysteresis loops, and the microscopic polarization reversal demonstrate the film has upward self-poling by PFM. The dielectric relaxation phenomenon (temperature around 270~300 K) was observed in the dielectric temperature and frequency spectrums and dielectric spectrum. The charge-related interfacial effect is the dominated factor and the intrinsic magnetoelectric coupling mechanisms can be excluded. The dielectric relaxation at high frequency may be related to not only MW effect but also Debye relaxation. Moreover, the electrical transport of LSMO film further proves that the dielectric relaxation behavior of the composite film is related to the resistance of LSMO due to the carrier accumulation at the junction surface. Our results of LSMO/BTO multiferroic composite films is very important for the applications of multiferroic materials in multi-state memory and other magnetoelectric fields.

## Data Availability

The data presented in this study are available on request from the corresponding author.
